# Insecticide resistance, fitness and susceptibility to Zika infection of an interbred *Aedes aegypti* population from Rio de Janeiro, Brazil

**DOI:** 10.1186/s13071-020-04166-3

**Published:** 2020-06-08

**Authors:** Carlucio Rocha dos Santos, Cynara de Melo Rodovalho, Willy Jablonka, Ademir Jesus Martins, José Bento Pereira Lima, Luciana dos Santos Dias, Mário Alberto Cardoso da Silva Neto, Georgia Correa Atella

**Affiliations:** 1 Laboratório de Sinalização Celular Programa de Biologia Molecular e Biotecnologia, Instituto de Bioquímica Médica Leopoldo de Meis, Universidade Federal do Rio de Janeiro, Rio de Janeiro, RJ Brazil; 2 Laboratório de Fisiologia e Controle de Artrópodes Vetores, Instituto Oswaldo Cruz, Fiocruz, Rio de Janeiro, RJ Brazil; 3 Laboratório de Entomologia, Instituto de Biologia do Exército, Rio de Janeiro, RJ Brazil; 4 Laboratório de Bioquímica de Lipídios e Lipoproteínas, Programa de Biologia Molecular e Biotecnologia, Instituto de Bioquímica Médica Leopoldo de Meis, Universidade Federal do Rio de Janeiro, Rio de Janeiro, RJ Brazil

**Keywords:** *Aedes aegypti*, Mosquito, Insecticide resistance, Fitness

## Abstract

**Background:**

*Aedes aegypti* is a vector of high relevance, since it transmits several arboviruses, including dengue, chikungunya and Zika. Studies on vector biology are usually conducted with laboratory strains presenting a divergent genetic composition from field populations. This may impair vector control policies that were based on laboratory observations employing only long maintained laboratory strains. In the present study we characterized a laboratory strain interbreed with *Ae. aegypti* collected from five different localities in Rio de Janeiro (*Aedes* Rio), for insecticide resistance (IR), IR mechanisms, fitness and Zika virus infection.

**Methods:**

We compared the recently established *Aedes* Rio with the laboratory reference strain Rockefeller. Insecticide resistance (deltamethrin, malathion and temephos), activity of metabolic resistance enzymes and *kdr* mutation frequency were determined. Some life table parameters (longevity, blood-feeding, number and egg viability) and Zika virus susceptibility was also determined.

**Results:**

*Aedes* Rio showed resistance to deltamethrin (resistance ratio, RR_50_ = 32.6) and temephos (RR_50_ = 7.0) and elevated activity of glutathione S-transferase (GST) and esterases (*α*-EST and *p*NPA-EST), but not acetylcholinesterase (AChE). In total, 92.1% of males genotyped for *kdr* presented a “resistant” genotype. Weekly blood-fed females from both strains, presented reduced mortality compared to sucrose-fed mosquitoes; however, *Aedes* Rio blood-fed females did not live as long (mean lifespan: Rockefeller = 70 ± 3.07; *Aedes* Rio = 53.5 ± 2.16 days). There were no differences between strains in relation to blood-feeding and number of eggs, but *Aedes* Rio eggs presented reduced viability (mean hatch: Rockefeller = 77.79 ± 1.4%; *Aedes* Rio = 58.57 ± 1.77%). Zika virus infection (plaque-forming unit, PFU) was similar in both strains (mean PFU ± SE: *Aedes* Rio: 4.53 × 10^4^ ± 1.14 × 10^4^ PFU; Rockefeller: 2.02 × 10^4^ ± 0.71 × 10^4^ PFU).

**Conclusion:**

Selected conditions in the field, such as IR mechanisms, may result in pleiotropic effects that interfere in general physiology of the insect. Therefore, it is important to well characterize field populations to be tested in parallel with laboratory reference strains. This practice would improve the significance of laboratory tests for vector control methods.
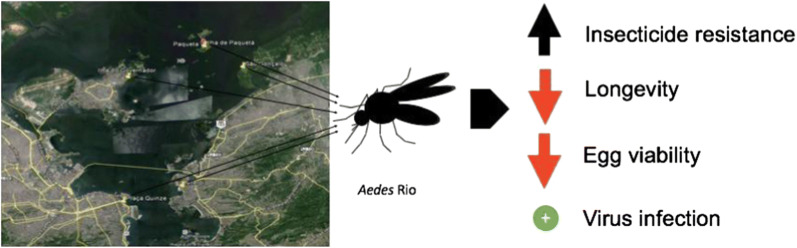

## Background

The mosquito *Aedes* (*Stegomyia*) *aegypti* (Linnaeus, 1762) is a cosmotropical species distributed through tropical and subtropical regions near human settlements [1–3]. This mosquito displays significant medical importance as a vector of several arboviruses, such as dengue virus, causing the most predominant arthropod-borne viral disease that affects humans [4]. Besides dengue virus, *Ae*. *aegypti* also transmits chikungunya, yellow fever and Zika viruses. The availability of only symptomatic treatment and the absence of vaccines for most of the aforementioned arboviruses, has led to the dependence of current disease control policies on vector control. However, sociocultural issues and economic problems impair infrastructure investments, a key approach to mosquito control, leading public policies to rely mainly on synthetic insecticides. Their overuse, however, may result in increased vector insecticide resistance, reducing the efficacy of this strategy [5, 6].

Several studies have been carried out aiming at improving the knowledge about vector biology, development of new classes of insecticides and novel methods of control, considering either suppression of population densities or their substitution for strains not able to transmit arboviruses [7]. Laboratory research on vectors is essential for understanding their role in pathogen proliferation and transmission, as well as for the development of enhanced vector control strategies [8, 9]. Studies underlining such assessments usually rely on strains long maintained in laboratory colonies. This is very convenient, since such strains eventually become highly adapted to laboratory conditions and are thus easily maintained. In addition, they can be shared among different laboratories and employed as a reference for reproducibility when standard conditions are followed. On the other hand, colonization and inbreeding processes lead to genetic bottlenecks, with consequent decreases in heterozygosity, as observed by microsatellite allelic richness analyses [10]. The pressure for laboratory adaptation and loss of characteristics selected for specific conditions in the field may result in the overall genetic composition, and consequently of diversified phenotypic traits, distinct from those of field populations [11–17]. Such phenotypic differences lead to differential fitness under a controlled environment, resulting in different responses in comparison to field populations [18–22]. This can be observed with insecticide resistance populations, which generally display reduced fitness under laboratory conditions, due to selected physiological changes on structural molecules or because displacement of energy for increased production of protective molecules against those chemicals [23].

Indeed, resistance to insecticides, such as temephos and DDT, led to a significant reduction of fertility life-table, blood-feeding and survival of *Ae. aegypti* and, ultimately, interfere with its vectorial capacity, a condition estimated through biological, ecological and behavioral parameters [24–26]. Moreover, field *Ae. aegypti* challenged with dengue virus present a significant infection rate variability compared to laboratory-reared strains, highlighting that field selection can also influence mosquito vector competence [8, 27].

Assessments concerning the development and reproduction aspects of vector populations have the potential to generate important data for combat policies and new control studies, leading to more accurate results. However, few studies have accessed the overall fitness aspects of field populations when evaluating vector control strategies [9, 26, 28]. Therefore, the present study aims to evaluate the insecticide resistance profile, fitness traits and vector competence to Zika virus in a field-representative *Ae. aegypti* population from Rio de Janeiro State, in comparison to a laboratory mosquito strain.

## Methods

### *Aedes* Rio population

The establishment of the *Aedes* Rio population was carried out with ovitraps randomly installed on house grounds across five localities in Rio de Janeiro (50 ovitraps in each location): Paquetá (22°49′27″S, 43°05′52″W), Niterói (22°53′14″S, 43°06′44″W), Praça XV (22°54′18″S, 43°10′19″W), São Gonçalo (22°46′45″S, 43°03′48″W) and Ilha do Governador (22°48′23″S, 43°11′46″W). Hatching of the eggs present on the paddles was induced in dechlorinated water containing 1 g of cat food (Friskies; Purina, Vevey, Switzerland). After adult emergence, *Ae*. *aegypti* mosquitoes were screened and separated by locality. Females were fed on blood in order to obtain eggs.

**Fig. 1:**
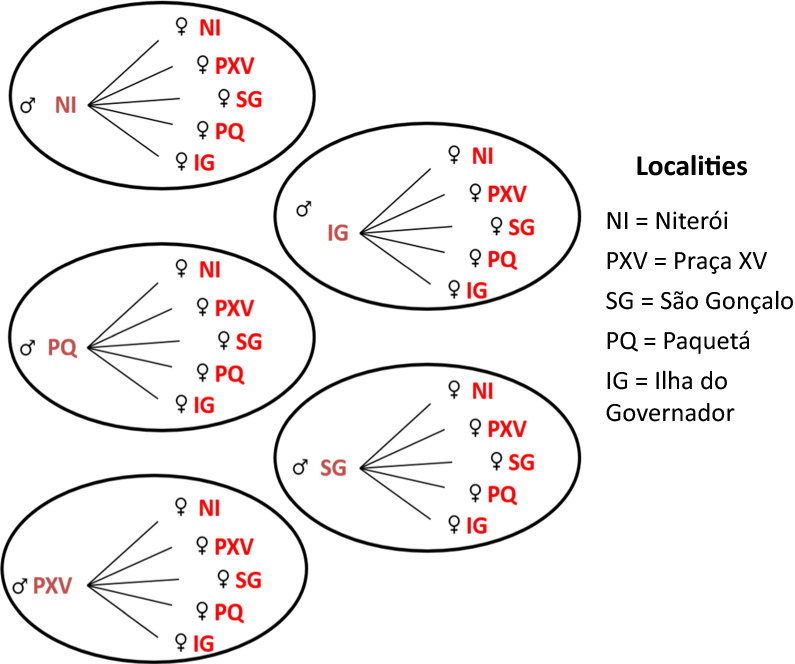
Mating scheme to obtain the *Aedes* Rio F1 population. A total of 25 cages were assembled, each one containing 20 males and 20 virgin females from each locality

Eggs from each locality were hatched and the larvae were raised as described previously. A total of 20 males and 20 virgin females from each location were placed in small cages to mate, totalling 25 cages (Fig. 1). After five days of free mating, females fed on blood and all mosquitoes were combined in a single cage in order to obtain a collective oviposition, generating *Aedes* Rio F1 eggs.

*Aedes* Rio F1 eggs were raised and two cages were assembled, each containing 500 males and 500 females. After 5 days of free mating, the females were fed on blood and all mosquitoes were combined in a single cage in order to obtain *Aedes* Rio F2 eggs. After the establishment of the *Aedes* Rio population, the following generations were used for the experiments.

### Mosquito rearing

All experiments were conducted with *Aedes* Rio F4, except for Zika virus (ZIKV) tests, which were performed with *Aedes* Rio F6. Comparisons were carried out with the Rockefeller strain, an widely used mosquito strain brought from the USA by the Brazilian Ministry of Health in the 1990s and distributed to Brazilian laboratories; since then, this strain has been used for insecticide tests and physiological studies.

The eggs were allowed to hatch for 2 h and approximately 300 larvae were reared in plastic trays containing 1 l of dechlorinated water and 1 g of cat food (Friskies; Purina). The food was provided every three days until pupation. The pupae were separated in plastic 50 ml cups and placed in cardboard cages until adult emergence. The larvae were kept in a biological oxygen demand incubator (BOD) at 26.5 ± 1 °C, with a relative humidity of 70 ± 10% and a 12 h photoperiod. Adults were maintained in a controlled environment at 26 ± 1 °C and a relative humidity of 70 ± 10%.

### Insecticide resistance assays

Dose-response bioassays were performed for the determination of the resistance profile to the organophosphate temephos of *Aedes* Rio larvae and the adult resistance profile to the organophosphate malathion and the pyrethroid deltamethrin used in control policies. Mosquitoes from the susceptible Rockefeller strain were used as reference and resistance ratios (RR) were calculated. The assays were performed following the WHO guidelines [29, 30] with slight modifications, and the WHO tubes methodology containing insecticide-impregnated papers was applied for the adult evaluation. Briefly, for larvae, 20 third-stage larvae (L3) were placed in 50 ml plastic cups, totalling four replicates (80 exposed larvae) for each insecticide concentration. A total of 11 increasing concentrations (ranging from 0.012 to 0.072 mg/l) in a final volume of 100 ml per glass were applied, and four glasses without insecticide were used as the controls. After 24 h of insecticide exposure, mortality rates were observed and recorded. A total of three bioassays were performed on different days.

For the adult tests, insecticide-impregnated papers were prepared at the Laboratório de Fisiologia e Controle de Artrópodes Vetores, Laficave, Brazil. Two control tube papers were impregnated only with the silicone vehicle. The papers were impregnated with insecticides at 10 different concentrations, as follows: malathion ranged from 0.05 to 0.5 g/m^2^ and deltamethrin, from 10.4 to 114.8 mg/m^2^. Three replicates were evaluated for each concentration. A total of 20 non-blood-fed females 3–5 days-old were transferred to each tube. All females remained in the test tubes for 1 h. After this period, the females were transferred and maintained in resting tubes (without any insecticide) for 24 h and mortality rates were observed and recorded. The assays were repeated three times, on different days. All bioassays were performed under controlled temperature and humidity conditions (26 ± 2 °C and 70 ± 10%, respectively), and a sugar solution was offered during the period the organisms remained in the resting tubes.

### Enzymatic assays

The activity of the main enzymes related to metabolic resistance, glutathione-S-transferases (GST), mixed function oxidases (MFO) and esterases (EST), were evaluated in one-day-old adult females and males. The procedures and analysis used are described in Valle et al. [31] and Viana-Medeiros et al. [32]. Briefly, the enzymatic assays were performed in duplicate in 96-well microtiter plates. Concerning esterases, the substrates α-naphthyl, β-naphthyl and *p*-nitrophenyl acetate (herein referred to as α-EST, β-EST and *p*NPA-EST) were employed. MFO was indirectly measured, whereas for acetylcholinesterase (AChE), both total activity and activity inhibited by propoxur were assayed. To calculate specific enzymatic activities, the total protein content of each specimen was quantified using a Bio-Rad protein assay/dye reagent concentrate (Bio-Rad, Hercules, USA). Standard susceptible profiles were taken from Rockefeller strain values. Mosquitoes from this reference strain were also included on all plates as an internal control. Enzyme activities were classified according to previously established criteria [33], as follows: after calculating the 99th percentile for the Rockefeller strain, the rate of specimens above this value was estimated for each enzyme and population. Activities were classified as unaltered, altered or highly altered when this rate was determined as < 15%, at 15–50 or > 50%, respectively.

### Molecular assay

DNA of 38 males was individually extracted. The mosquitoes were homogenised in 500 µl of a TNES buffer (Tris 50 mM, NaCl 400 mM, EDTA 20 mM and SDS 0.5%) and incubated with 0.2 mg/l proteinase K at 56 °C for 3 h. Then, precipitations and alcohol washes were performed, as described in Martins et al. [34].

SNP genotyping assay was used to verify the presence of knockdown resistance (*kdr*) mutations 1016 (Val^+^ and Ile^*kdr*^) and 1534 (Phe^+^ and Cys^*kdr*^) in the voltage-gated sodium channel gene (*AaNa*_*V*_). The customized TaqMan Genotyping Assay method (Thermo Fisher Scientific, Waltham, USA) was performed in independent reactions for each site. Primers and probes are listed in Table 1. The reactions were prepared in 10 µl, containing 1 µl of DNA (~ 10 ng), 1× TaqMan Genotyping Master Mix and TaqMan Assay, combining 0.5× of primers and 1× of probes for the 1534 and 1016 sites, in a 96-well microplate. As positive controls, the Rockefeller strain (SS), Rock-*kdr* strain (RR) [35], and a mix with equimolar amounts of Rockefeller and Rock-*kdr* DNA (RS) were used. The thermocycling conditions followed the manufacturer instructions (TaqMan Genotyping Assay; Thermo Fisher Scientific) and the reactions were conducted using a real time QuantStudio 6 thermocycler (Thermo Fisher Scientific). As described elsewhere [36], the allelic and genotypic frequencies considered the 1016 and 1534 sites as a unique locus, and three alleles were constituted as Na_V_S (1016 Val^+^ + 1534 Phe^+^), Na_V_R1 (1016 Val^+^ + 1534 Cys^*kdr*^), and Na_V_R2 (1016 Ile^*kdr*^ + 1534 Cys^*kdr*^).

**Table 1:** Primers and probes used in the SNP genotyping assay reactions

Primer sequence (5′-3′)	Probes (5′-3′)
1016for: CGTGCTAACCGACAAATTGTTTCC	1016Val^+^ VIC-CCGCACAGATACTTA-NFQ
1016rev: GACAAAAGCAAGGCTAAGAAAAGGT	1016Ile^kdr^ FAM-CCCGCACAGGTACTTA-NFQ
1534for: CGAGACCAACATCTACATGTACCT	1534Phe^+^ FAM-ACGACCCGAAGATGA-NFQ
1534rev: GATGATGACACCGATGAACAGATTC	1534Cys^kdr^ VIC-AACGACCCGCAGATGA-NFQ

Hardy-Weinberg equilibrium was assessed by the classical equation [37], the null hypothesis of equilibrium was checked by a Chi-square test with three degrees of freedom.

### Viral infection

Zika viral stocks (Uganda strain) were propagated in C6/36 cells maintained in a Leibovitz-15 medium supplemented with 10% fetal bovine serum, 1% penicillin/streptomycin, 1% fungizone and HEPES buffer 2 mM. Culture supernatants containing viral particles were harvested, centrifuged at 4500× *g* for 10 min at 4 °C, aliquoted, and stored at − 70 °C until use. Viral titers were determined by a plaque assay as 2 × 10^7^ PFU/ml. Plastic cages containing 200 7 days-old *Ae*. *aegypti* mosquitoes (100 males and 100 females) were artificially fed with a 1:1 solution of heparinized and washed rabbit erythrocytes and DMEM culture medium containing ZIKV (final titer 1 × 10^7^ PFU/ml). Feeding was performed using water-jacketed artificial feeders maintained at 37 °C and sealed with parafilm membranes for approximately 1 h inside a Biosafety level 2 (BSL-2) insectary facility. The insects were starved for 12 h prior to feeding. Unfed mosquitoes were removed from the cages in all the experiments. Mosquitoes were then maintained under BSL-2 insectary conditions (28 °C, L:D 12:12 h, 70% humidity) with access to a 10% sucrose solution. Females were then individually transferred to cryotubes 7 days post-blood meal and stored at − 70 °C until assay.

A plaque assay was performed, for viral load evaluation, as previously described [38]. Briefly, Vero cells were cultured in complete DMEM media, supplemented with 10% fetal bovine serum, 1% penicillin/streptomycin, 1% l-glutamine, 1% fungizone and HEPES buffer 2 mM. One day prior to the assay, the cells were plated into 24-well plates at 70–80% confluence. The mosquitoes were homogenized using a homogenizer, centrifuged at 4000× *g* for 10 min at 4 °C and four serial dilutions (10-fold) were performed. Each dilution was inoculated in a single well. The plates were then gently rocked for 15 min at RT and incubated for 45 min at 37 °C and 5% CO_2_. Finally, an overlay of DMEM containing 0.8% methylcellulose and 10% FBS was added to each well, and the plates were incubated for 5 days. To fix and stain the plates, the culture media was discarded and a 1:1 (v:v) methanol and acetone solution and 1% crystal violet was used. The plaque-forming units (PFU) were counted and corrected by the dilution factor. Mosquito viral loads (whole body PFU) and infection rates (IR: percentage of infected mosquitoes) were evaluated.

### Developmental and reproductive parameter evaluation

#### Adult longevity

Newly emerged adults (less than 24 h post-emergence from the pupae exuviae) were placed in small cylindrical cardboard cages containing 15 couples and fed one of two food diets: (i) two cages received sugar solution *ad libitum* as the only food source; and (ii) two cages, in addition to the sugar solution, received blood meals once a week (with prior 24 h withdrawal of the sugar solution); in this case, anesthetized guinea pigs were offered for blood-feeding for 30 min. Mortality was scored every day until all mosquitoes were dead. This assay was performed four times.

#### Blood-feeding

The amount of ingested blood was assessed with approximately 5 days-old females. Mosquitoes were deprived of the sugar solution 24 h before the assay. Four pools of 10 females each were killed by ethyl acetate exposure and weighed on an analytical balance (APX - 200; Denver Instrument, New York, USA). Anesthetized guinea pigs were offered to another group of live females and, after 30 min, an additional four groups of 10 fully engorged females were killed and weighed as described above. This assay was performed four times on different days.

#### Oviposition and egg viability

Oviposition was carried out three days after blood-feeding. Briefly, around 40 *Aedes* Rio females and 40 Rockefeller strain females were individually transferred to small Petri dishes (9 cm in diameter) lined with filter paper on their lids. After moistening the filter paper with 3 ml dechlorinated water, the Petri dishes remained in a BOD incubator with humidity set at *c.*70%; after two days, the number of egg-laying females and eggs were recorded. After the eggs had dried and were counted, 10 ml of dechlorinated water was added to each plate. The eggs were allowed to hatch for two days and hatching larvae were counted. This assay was performed three times during different periods.

### Statistical analyses

Lethal concentrations (LCs) were calculated using the Probit analysis available in the Polo-PC statistic package [39] and the RR were calculated by the division of the *Aedes* Rio LCs and the respective Rockefeller strain LCs. The populations were classified according to the criteria adopted by the WHO [30], in which populations with RR_50_ < 5 are considered susceptible, RR_50_ between 5 and 10 are considered populations displaying moderate resistance and RR_50_ > 10 are considered highly resistant.

Allelic and genotypic *kdr* frequencies were calculated as described elsewhere [36]. The number of “resistant” genotypes was estimated as the sum of the homozygous and heterozygous *kdr* genotypes (R1R1, R2R2 and R1R2) [40].

Lifespan comparisons were carried out by survivor curve and Mantel-Cox and Gehan-Breslow-Wilcoxon test calculations. Data normality was tested using the Shapiro-Wilk W test prior to additional statistical analyses. When data displayed a normal distribution, comparisons were performed by an unpaired Studentʼs t-test (*P* < 0.05) for female weight, blood intake and blood-feeding. Non-parametric tests were applied for non-normal distributions and differences and data comparisons for oviposition, egg viability and viral susceptibility, which were assessed by the Mann-Whitney U-test (*P* < 0.05). Plots and analyses were performed using GraphPad Prism 5 software (GraphPad, La Jolla California, USA).

## Results

### Insecticide resistance profile of the *Aedes* Rio population

#### Insecticide bioassay

**Fig. 2:**
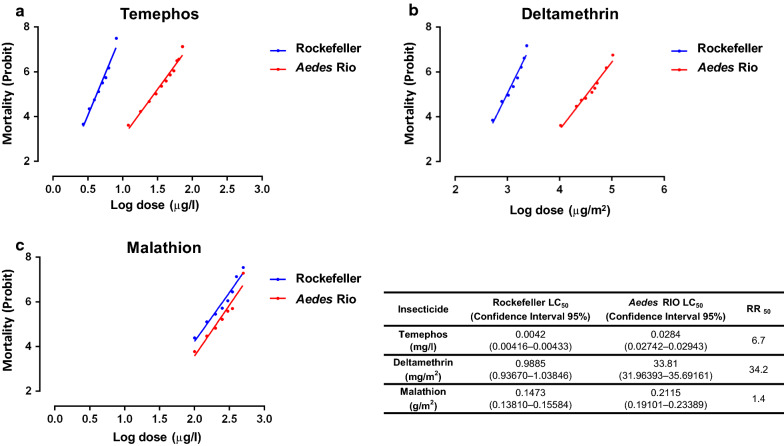
Linear regression for *Aedes* Rio mortality after exposure to temephos (**a**), deltamethrin (**b**) and malathion (**c**). Mosquitoes from the Rockefeller strain were used for comparison in all tests (blue lines)

The insecticide resistance profile of *Aedes* Rio larvae exposed to temephos and adults exposed to deltamethrin and malathion exposures were determined (Table 2, Fig. 2). Dose response bioassays indicated that *Aedes* Rio larvae presented moderate insecticide RR to temephos (LC_50_ = 0.028 mg/l; RR_50_ = 6.6). *Aedes* Rio female adult mosquitoes presented high insecticide RR to deltamethrin (LC_50_ = 32.25 mg/m^2^; RR_50_ = 33.1) but were susceptible to malathion (LC_50_ = 0.207 g/m^2^; RR_50_ = 1.4). When comparing the slopes, *Aedes* Rio presented greater heterogeneity than the Rockefeller strain (lower slope values).

**Table 2:** Resistance profile of *Aedes* Rio mosquitoes to temephos, deltamethrin and malathion

Insecticide	Population	LC_50_	95% CI LC_50_	LC_95_	95% CI LC_95_	Slope	RR_50_^a^	RR_95_
Temephos	Rockefeller	0.0042	0.0026–0.0066	0.0067	0.0041–0.011	8.0	1.0	1.0
	*Aedes* Rio	0.028	0.0206–0.038	0.068	0.045–0.102	4.3	6.6	10.1
Deltamethrin	Rockefeller	0.974	0.59–1.61	2.17	1.1466–4.09	4.7	1.0	1.0
	*Aedes* Rio	32.25	21.1–49.3	100.9	53.60–189.93	3.3	33.1	46.6
Malathion	Rockefeller	0.150	0.076–0.299	0.34	0.207–0.564	4.6	1.0	1.0
	*Aedes* Rio	0.207	0.108–0.396	0.48	0.199–1.17	4.5	1.4	1.4

^a^*Aedes* Rio presenting RR_50_ < 5 were considered susceptible, RR_50_ between 5 and 10 were considered moderately resistant and RR_50_ > 10 were considered resistant

*Notes*: lethal concentration (LC) values expressed as mg/l, mg/m^2^ and g/m^2^, respectively. Confidence intervals (CI) of lethal concentrations, slope and resistance ratio (RR) 50 and 95 are also shown

*Abbreviations*: LC50, concentration that kills 50% of the animals; LC95, concentration that kills 95% of the animals

#### Enzymatic assay

Since insecticide resistance is usually related to metabolic resistance, detoxification enzyme activities, involved with altered RR [41], were also assayed (Table 3, Additional file 1: Figure S1). In comparison to the Rockefeller strain, *Aedes* Rio males and females presented altered *α*-EST activity (*H* = 67.54, *df* = 3, *P* < 0.0001) and highly altered GST activity (*H* = 137.7, *df* = 3, *P* < 0.0001). Altered female and highly altered male *p*NPA-EST activities were also observed (*H* = 66.33, *df* = 3, *P* < 0.0001). No difference was observed for MFO activity, although *Aedes* Rio presented lower *β*-EST activities (*H* = 59.87, *df* = 3, *P* < 0.0001) (Additional file 1: Figure S1).

**Table 3:** Enzyme activity alterations in *Aedes* RIO males and females compared to the Rockefeller strain

*Aedes* Rio	AChE	GST	α-EST	β-EST	*ρ*NPA-EST	MFO
Male	0	***76***	*28*	0	***55***	0
Female	10	*50*	*16*	0	*28*	0

*Notes*: Enzyme activities were classified as unaltered (regular font), altered (italic) and highly altered (italic and bold), if < 15, 15–50 or > 50% of individuals, respectively, presented activities above the corresponding Rockefeller 99th percentile value (see “Methods” for more details)

*Abbreviations*: MFO, multi-function oxidases; EST, esterases; α-EST, β-EST and *ρ*NPA-EST, substrates (α- and β-naphthyl and *ρ*-nitrophenil acetates) employed for EST, GST, glutathione S-transferase

#### Molecular assay

Target site insensitivity due to mutation is another insecticide resistance mechanism found in several field populations. For this reason, the frequency of *kdr* mutations at the 1016 and 1534 sites in the IIS6 and IIIS6 *AaNa*_*V*_ segments, respectively, were investigated in 38 males. The genotype frequencies did not deviate from the Hardy-Weinberg assumption (*χ*^2^ = 1.73, *P* = 0.3298), suggesting that the *kdr* alleles reached an equilibrium in this strain and that the genotypes are not under any evident selection in laboratory conditions. The results analysed for both sites in linkage and as an individual locus [36] are provided in Table 4.

**Table 4:** Allelic and genotypic frequencies of the *Aedes* Rio population for the alleles Na_V_S

Population	Allelic frequency			Genotypic frequency						“Resistant” genotypes^a^
	S	R1	R2	SS	SR1	R1R1	SR2	R1R2	R2R2	
*Aedes* Rio	0.039	0.539	0.422	0	0.026	0.263	0.053	0.526	0.132	0.921

^a^R1R1 + R1R2 + R2R2

*Notes*: Na_V_S (1016 Val^+^ + 1534 Phe^+^), Na_V_R1 (1016 Val^+^ + 1534 Cys^*kdr*^) and Na_V_R2 (1016 Ile^*kdr*^ + 1534 Cys^*kdr*^)

The alleles Na_V_R1 and Na_V_R2 were present at high frequencies. The SS genotype was not detected and “resistant genotypes” were observed at a very high frequency (92.1%).

### Fitness evaluation of *Aedes* Rio

#### Adult lifespan

**Fig. 3:**
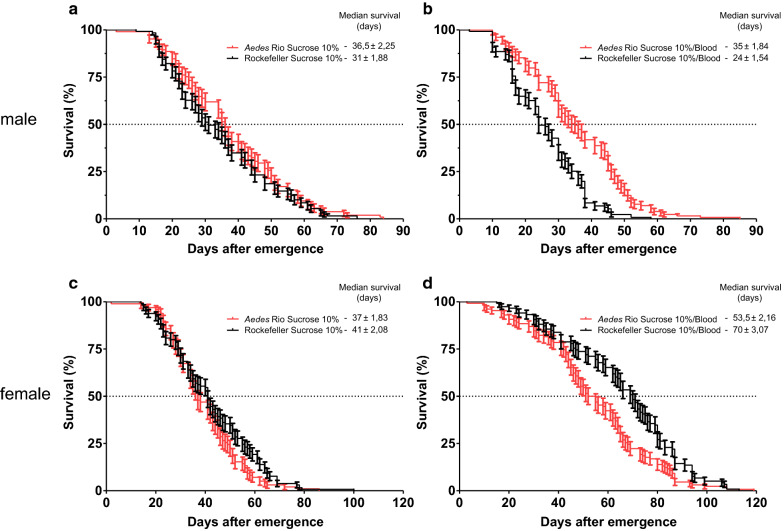
Lifespan under two different feeding regimens for Rockefeller and *Aedes* Rio strains: **a**, **c** Mosquitoes in cages under a 10% sucrose diet (**a**, males; **c**, females). **b**, **d** Mosquitoes in cages with blood offered weekly in addition to 10% sucrose (**b**, males; **d**, females). Each curve represents the mean of four independent experiments with fifteen couples. Error bar: standard error of the mean (SE)

No significant differences in male and female lifespans between both populations in cages under 10% sucrose *ad libitum* were observed (Fig. 3; male median survival (median ± SEM): Rockefeller (31 ± 1.88 days), *Aedes* Rio (36.5 ± 2.25 days), Mantel-Cox test *P* = 0.2088; female median survival (median ± SEM): Rockefeller (41 ± 2.08 days), *Aedes* Rio (37 ± 1.83 days), *P* = 0.061). However, in the cages with blood-feeding offered on a weekly basis, Rockefeller males presented reduced survival in comparison to *Aedes* Rio males (median survival, in days (median ± SEM): Rockefeller: 24 ± 1.54; *Aedes* Rio: 35 ± 1.84, *P* < 0.0001) and Rockefeller males from cages with 10% sucrose offered *ad libitum* (*P* < 0.0001). However, blood-fed Rockefeller females presented longer lifespans compared to *Aedes* Rio females (median survival, in days (median ± SEM): Rockefeller: 70 ± 3.07; *Aedes* Rio: 53.5 ± 2.16, *P* < 0.0003). Females from both populations offered blood on a weekly basis also presented reduced mortality in comparison to sucrose-fed females (Rockefeller *P* < 0.0001; *Aedes* Rio *P* < 0.0001).

#### Weight and blood-feeding

**Fig. 4:**
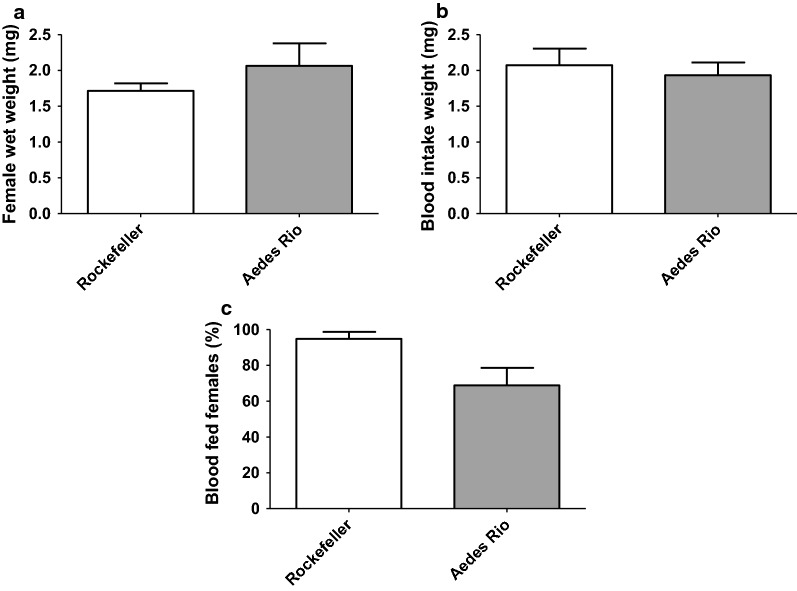
Female weight and blood-feeding for the Rockefeller strain and the *Aedes* Rio population. Female weight (**a**), ingested blood weight (**b**), percentage of blood-fed females (**c**). Data represent the means of four independent experiments, each consisting in four pools containing 10 females. Blood weight was obtained by subtraction of the weight of unfed from the weight of blood-fed female pools. Error bar: standard error of the mean (SE)

For estimates on the amount of blood taken, pools of insects before and after their blood meal were weighed. The weight before the blood meal also served as an estimate for whole-body size. No significant difference in female mosquito weight (Fig. 4a; mean ± SE: Rockefeller: 1.72 ± 0.11 mg; *Aedes* Rio: 2.06 ± 0.31 mg, ANOVA: *F* = 8.83 *P* = 0.3325) and the total amount of ingested blood (Fig. 4b; mean ± SE: Rockefeller: 2.07 ± 0.23 mg; *Aedes* Rio = 1.93 ± 0.18 mg, ANOVA: *F* = 1.64 *P* = 0.6461) were observed between the Rockefeller strain and the *Aedes* Rio population.

No difference between the two populations concerning the total number of blood-fed females was observed (Fig. 4c; mean ± SE: Rockefeller: 94.8 ± 4.0%; *Aedes* Rio: 68.8 ± 9.8%, Mann Whitney test *U* = 1.0, *Z* = 2.19, *P* = 0.057).

#### Female fertility

**Fig. 5:**
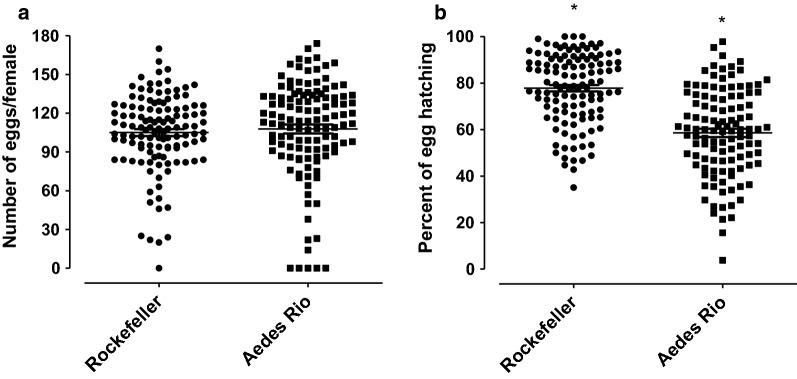
Oviposition and egg viability comparison between the Rockefeller strain and the *Aedes* Rio population. **a** Number of eggs per female. **b** Egg viability. Data represent three independent experiments, each consisting of 40 females. Each dot indicates the number of eggs or egg viability from an individual female. Error bar: SE

The total number of eggs/female and the rate of egg hatching are shown in Fig. 5. A comparison between populations revealed no difference in the total number of laid eggs (Fig. 5a; mean ± SE: Rockefeller: 105.1 ± 2.72; *Aedes* Rio: 107.8 ± 3.49, Mann Whitney test *U* = 6466, *Z* = 1.37, *P* = 0.1723). However, the *Aedes* Rio population showed lower egg viability in comparison to the Rockefeller strain (Fig. 5b; mean ± SE: Rockefeller: 77.79 ± 1.4%; *Aedes* Rio: 58.57 ± 1.77%, Mann Whitney test *U* = 2884, *Z* = 7.45, *P* < 0.0001).

#### Virus susceptibility

**Fig. 6:**
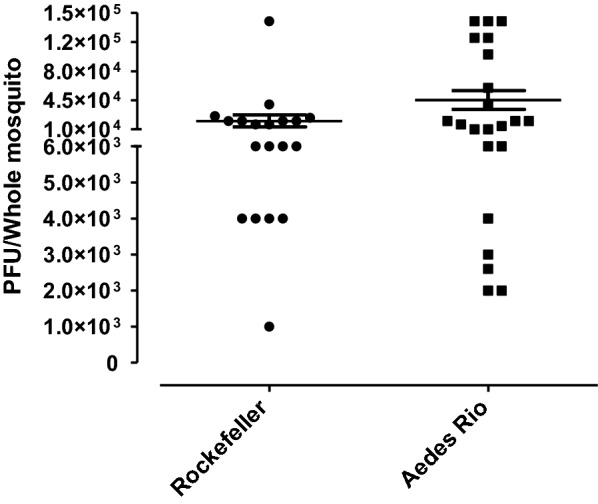
Mosquito viral susceptibility. Females were fed viremic blood containing 10^7^ PFU/ml of the Zika virus, and the number of PFU was determined in whole mosquitoes (viral load) at seven days post-infection. Error bar: SE

Susceptibility to Zika infection in mosquitoes artificially fed viremic blood meals was assessed (Fig. 6). The *Aedes* Rio population showed similar Zika viral loads and infection rates (mean ± SE: 4.53 × 10^4^ ± 1.14 × 10^4^ PFU; IR: 100%) compared to the Rockefeller strain (mean ± SE: 2.02 × 10^4^ ± 0.71 × 10^4^ PFU; IR: 100%, Mann Whitney test *U* = 176, *Z* = 0.25, *P* = 0.3929).

## Discussion

Distinct field mosquito populations may diverge from laboratory-reared mosquitoes in regard to the overall fitness, especially when they possess a distinct profile for resistance to insecticides, as vastly documented [26, 27]. Still, even when similar phenotypes are observed between field and laboratory strains, these similarities may have different genetic sources. For example, the comparison between profiles of field insecticide-resistant mosquitoes and organophosphate-selected laboratory strains presented a similar temephos resistance ratio. However molecular analysis revealed different detoxification enzyme patterns with the presence of different classes of enzymes responsible for insecticide resistance between populations, revealing that the ecosystem may select different detoxification mechanisms [25]. Therefore, careful fitness evaluations concerning *Ae. aegypti* mosquitoes with a similar genetic profile to field populations may result in relevant information for vector control policies, such as the selection of a suitable insecticide class or other strategies, since fitness aspects of natural populations may cause diversified responses in comparison to those observed in a reference laboratory strain.

*Aedes* Rio insecticide resistance was observed against temephos and deltamethrin, but not malathion. To date, no mutations in the acetylcholinesterase gene (*ace-1*) leading to organophosphate resistance have been described in *Ae*. *aegypti*. In this way, resistance to this insecticide is mainly due to metabolic changes. This type of resistance may be diverse in the different mosquito life stages (temephos-larvicide; malathion-adulticide) which may impair cross-resistance mechanisms and stage-related overall fitness, since these enzymes also play a role in physiological functions. The extensive use of insecticides in vector control in several Brazilian states has led to increased mosquito resistance to temephos and deltamethrin. Indeed, some studies have reported increasing *Aedes* resistance to the organophosphate temephos and pyrethroids in Brazil recently, especially in coastal areas [36, 42–44]. Studies evaluating *Ae. aegypti* populations from Rio de Janeiro have reported high insecticide resistance in several regions [33, 36, 40, 45–48]. Therefore, the insecticide resistance profile observed herein in *Aedes* Rio must have been inherited from the field populations used for interbreeding.

Usually, high insecticide RR levels are associated with metabolic changes such as higher expression or enhanced efficiency of detoxification enzymes [41, 49]. These enzymes display a broad substrate spectrum with variable affinities and are, therefore, able to detoxify a wide range of insecticide classes [43]. In the present study, no difference was observed for AChE activity in *Aedes* Rio when compared to the Rockefeller strain. However, metabolic detoxification changes were noted, mainly, for GST, *α*-EST and *p*NPA-EST, which showed higher enzyme activities, but not *β*-EST. These enzymes have previously been noted as exhibiting enhanced activity after organophosphate selection and are associated with organophosphate resistance [25, 33, 46, 50–54]. Diniz et al. [25] observed higher GST and esterase activities in a Brazilian laboratory strain under temephos selection, similar to field populations. Similar results were reported by Bellinato et al. [43] when evaluating several Brazilian *Ae. aegypti* populations showing higher esterase and GST activities than MFO enzymes.

Several studies have correlated MFO activity or expression levels of the *P450 cyp* family genes to increased pyrethroid resistance; however, so far there is no particular marker available for large-scale analysis [55–58]. On the other hand, genotyping of specific *kdr* mutations has also been well correlated to pyrethroid resistance, and are thus currently widely used in molecular surveillance of insecticide resistance [28, 59–64]. No altered MFO concentrations were observed herein. However, a high frequency of “resistant” *kdr* genotypes (92.1%) was noted, suggesting that deltamethrin resistance is primary due to target site alteration and not to metabolic mechanisms. In *Ae. aegypti*, two mutations in the V1016G/I site and one in F1534C site have been observed in several resistant field populations worldwide [65–67]. Reports on Brazilian *Ae. aegypti* populations indicate a high frequency of both V1016I and F1534C substitutions in comparison to susceptible alleles [36, 40, 64, 68, 69]. Thus, the enzymatic activity variations and *kdr* mutations observed in *Aedes* Rio were inherited characteristics from the mosquito populations collected from Rio de Janeiro and used for interbreeding, leading to the enhanced RR observed herein.

The impact of insecticide resistance on the overall fitness of mosquitoes under an environment free of insecticides is a known phenomenon, and involves several negative effects on development and reproduction [25, 26, 35, 62, 70, 71]. This is of epidemiological relevance since resistance tends to be lost along generations in the absence of insecticide selection. Therefore, in addition to understanding the status of susceptibility to the current method employed against a target vector, it is also desirable to unravel which alterations might have been selected in its life table. In this study, we did not observe lifespan differences between males of both Rockefeller and Rio strains from cages with a constant source of sucrose. Notwithstanding, Rockefeller males maintained in cages with blood offered on a weekly basis had lower survival rates compared to Rockefeller and *Aedes* Rio males kept in cages under a sucrose diet only. This may be related to the experimental design in which the sucrose-soaked cotton is withdrawn from the cage, leading to mosquito fasting which in turn results in greater female blood-feeding rates. Rockefeller males did not survive as long as *Aedes* Rio males when deprived from the sugar source. We hypothesize that although reared in parallel under the same laboratory conditions, these strains should have either acquired distinct nutrition reserves during the development or metabolized them differently. Accordingly, field-collected *Anopheles gambiae* males had a higher total lipid content than male mosquitoes of a laboratory strain reared under insectary feeding and optimum density conditions, which may be associated with differences in nutritional reserves and, therefore, fasting tolerance [72]. This higher tolerance for fasting periods in field mosquitoes is likely to be a consequence of distinct physiological traits between field and laboratory reference strains, which maintain field mosquitoes under more adverse conditions.

Rockefeller females showed higher survival rates under blood-feeding conditions compared to *Aedes* Rio females. Lifespan reports comparing *Ae. aegypti* laboratory strains and field populations are diverse. Some studies indicate similar longevity for field and laboratory mosquitoes [26, 70]. Other studies report reduced lifespans of field mosquitoes, usually due to high insecticide resistance ratios [19, 71]. For example, a *Culex pipiens* population resistant to organophosphates, likely due to increased esterase activity, had low survival rates [73]. Moreover, when resistance to temephos decreased in an *Ae. aegypti* field population, its longevity inversely increased [25]. In the present study, the lower survival rate of *Aedes* Rio females is probably one of the pleiotropic effects of mechanisms selected for resistance to insecticides, which may result in a high fitness cost under an environment free of insecticide, such as standard laboratory conditions.

Weekly blood-feeding led to increased female lifespan in both Rockefeller and *Aedes* Rio strains. Although it would be expected that blood-feeding would reduce the lifespan due to an increase in reactive oxygen species production and immune challenges [74–76], it is well documented that blood-feeding increases mosquito survival [35, 77–82]. A longer lifespan in blood-fed females as observed herein, may be related to a richer nutritional composition. A longer lifespan is beneficial for vector competence, since female mosquitoes have time for multiple blood-feedings and therefore an increased chance to become infected and transmit arboviruses and pathogens.

Blood meals are an important parameter, since they are related to vectorial capacity through parasite ingestion and the number of deposited eggs [26, 71]. Thus, compared to the Rockefeller strain, *Aedes* Rio showed no difference in the amount of blood ingested. Accordingly, previous studies observed that field populations resistant to insecticides, also did not show difference in the amount of ingested blood compared with reference strains [28]. The number of females that accepted a blood source offered in the laboratory was lower in insecticide-resistant field populations [23, 26]. This could be related to pleiotropic effects of mechanisms selected for resistance, or due to the laboratory conditions of blood-feeding to which the reference colonies are better adapted.

As the amount of blood ingested did not differ between the Rockefeller and Rio strains, the total number of oviposited eggs was similar between them. On the other hand, the fertility (rate of egg hatching) in *Aedes* Rio was lower than in *Aedes* Rockefeller. There are several examples evidencing the relationship between insecticide resistance status and reduced egg viability, as in *Ae*. *aegypti* populations resistant to permethrin [83] and temephos [25]. In contrast, several other studies have reported no difference in the viability of eggs from insecticide resistant populations [26, 35, 71]. This reinforces that diversified mechanisms selected for resistance in conjunction with the whole genetic background will determine the extension of interreference that those mechanisms will play in the overall insect phenotype.

In relation to vector competence, reports on *Ae. aegypti* susceptibility to the Zika virus are diverse, with varying viral loads and infection rates in this species [84–86]. In our study, *Aedes* Rio showed higher infection rates (85–90%) when compared to other field mosquitoes [87, 88]. For instance, laboratory infection rates varied between 40% in *Ae. aegypti* from Fernando de Noronha, PE, Brazil [85] and 60–80% in mosquito populations from Mexican cities [89]. These differences can be related to diverse factors, such as virus titre, virus strain and the mosquito genetic background [85, 87, 89–91].

## Conclusions

Altogether, the present study revealed that *Aedes* Rio, a laboratory interbred strain of *Ae. aegypti*, originating from field mosquito populations collected from Rio de Janeiro, is resistant to the insecticides temephos and deltamethrin. Metabolic and target site resistance mechanisms selected in the original populations may be responsible for pleiotropic effects that caused a fitness cost in an environment free of insecticides, when compared to the laboratory reference strain Rockefeller. This decreased fitness however did not result in avoiding Zika virus infection and dissemination in the *Ae. aegypti* Rio strain. We suggest that vector control programmes should consider adopting a recently established colony originating from the targeted population, in addition to a commonly employed laboratory reference strain. As observed here, diverse field-selected aspects, such as insecticide resistance, may significantly interfere with development and reproduction traits, which would potentially lead to uncertain conclusions when testing chemicals or new vector control strategies.

## Supplementary information

**Additional file 1: Figure S1.** Detoxification enzyme activity of one-day-old mosquitoes from the Rockefeller strain and the *Aedes* Rio F4 population. Symbols represent significant differences between the same gender by a non-parametric One-way ANOVA test (Kruskal-Wallis) with Dunn’s *post-hoc* test (*P* = 0.05%). The data represent two independent experiments totalling 50 mosquitoes for each condition. Mean deviation: SE. Statistical difference between populations (*P* < 0.05).

## Data Availability

All data generated or analysed during this study are included in this published article and its additional file.

## Abbreviations

*AaNa*_*V*_: voltage-gated sodium channel gene
*ace-1*: acetylcholinesterase gene
AChE: acetylcholinesterase
BOD: biological oxygen demand incubator
BSL: biosafety level
Cys: cysteine
EST: esterase
GST: glutathione S-transferase
Ile: isoleucine
IR: infection rate
*kdr*: knockdown resistance gene
LC: lethal concentration
MFO: mixed function oxidases
PFU: plaque-forming units
Phe: phenylalanine
SE: standard error of the mean
SEM: standard error of the median
Val: valine
ZIKV: Zika virus
