# Supplementary material for: Insecticide resistance, fitness and susceptibility to Zika infection of an interbred Aedes aegypti population from Rio de Janeiro, Brazil
Source: Parasit Vectors. 2020 Jun 8;13:293. doi: 10.1186/s13071-020-04166-3 (PMC7281914; doi:10.1186/s13071-020-04166-3)
Supplement: Supplementary file 1 — Additional file 1: Figure S1. Detoxification enzyme activity of one-day-old mosquitoes from the Rockefeller strain and the Aedes Rio F4 population. Symbols represent significant differences between the same gender by a non-parametric One-way ANOVA test (Kruskal-Wallis) with Dunn’s post-hoc test (P = 0.05%). The data represent two independent experiments totalling 50 mosquitoes for each condition. Mean deviation: SE. Statistical difference between populations (P < 0.05). [file 13071_2020_4166_MOESM1_ESM.pdf]

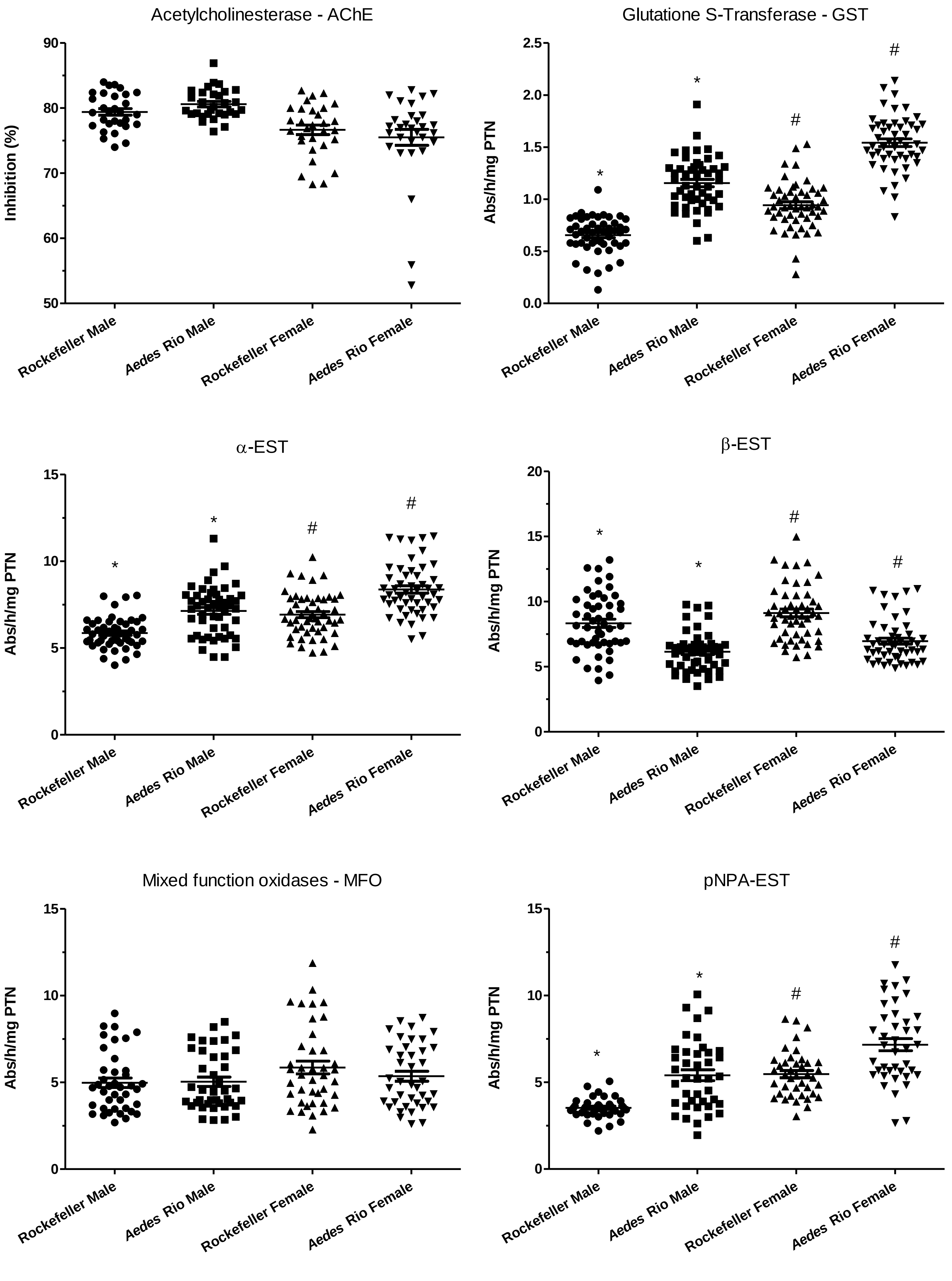

Acetylcholinesterase - AChE
Inhibition (%)
90
80
70
60
50
Rockefeller Male
Aedes Rio Male
Rockefeller Female
Aedes Rio Female
Glutatione S-Transferase - GST
Abs/h/mg PTN
2.5
2.0
1.5
1.0
0.5
0.0
*
*
#
#
Rockefeller Male
Aedes Rio Male
Rockefeller Female
Aedes Rio Female
α-EST
Abs/h/mg PTN
15
10
5
0
*
*
#
#
Rockefeller Male
Aedes Rio Male
Rockefeller Female
Aedes Rio Female
β-EST
Abs/h/mg PTN
20
15
10
5
0
*
*
#
#
Rockefeller Male
Aedes Rio Male
Rockefeller Female
Aedes Rio Female
Mixed function oxidases - MFO
Abs/h/mg PTN
15
10
5
0
Rockefeller Male
Aedes Rio Male
Rockefeller Female
Aedes Rio Female
pNPA-EST
Abs/h/mg PTN
15
10
5
0
*
*
#
#
Rockefeller Male
Aedes Rio Male
Rockefeller Female
Aedes Rio Female
